# Screening for Atrial Fibrillation using Economical and accurate TechnologY (SAFETY)—a pilot study

**DOI:** 10.1136/bmjopen-2016-013535

**Published:** 2017-01-13

**Authors:** Mark Lown, Arthur Yue, George Lewith, Paul Little, Mike Moore

**Affiliations:** 1 Primary Care and Population Sciences, Faculty of Medicine, University of Southampton, Aldermoor Health Centre, Southampton, UK; 2 Cardiology and Electrophysiology, Southampton General Hospital, Southampton, UK

## Abstract

**Introduction:**

Atrial fibrillation (AF) is a cause of stroke and a marker of atherosclerosis and of all patients with stroke, around 17% have AF. The screening and treatment of AF could prevent about 12% of all strokes. Several relatively low-cost devices with good accuracy now exist which can detect AF including WatchBP and AliveCor. However, they can only measure the ECG or pulse over short time periods. Inexpensive devices such as heart rate monitors, which are widely available, can measure heart rate for prolonged periods and may have potential in screening for AF. This study aims to determine the accuracy of AliveCor and WatchBP along with a bespoke algorithm using a heart rate monitor belt (Polar H7) and a wearable RR interval recorder (Firstbeat Bodyguard 2) for detecting AF during a single screening visit in primary care patients.

**Methods/analysis:**

A multicentre case–control diagnostic study comparing the four different devices for the detection of AF with a reference standard consisting of a 12-lead ECG in GP surgeries across Hampshire, UK. We aim to recruit 92 participants with AF and 329 without AF aged 65 years and over. We will ask participants to rate comfort and overall impression for each device. We will collect qualitative data from participants capturing their experience of using wearable devices in order to evaluate acceptability. We will collect data from GPs to determine their views on AF screening.

**Ethics and dissemination:**

This protocol was approved by the London—City & East Research Ethics Committee in June 2016. The findings of the trial will be disseminated through peer-reviewed journals, national and international conference presentations and the Atrial Fibrillation Association, UK.

**Trial registration number:**

ISRCTN17495003, Pre-results.

**Strengths and limitations of this study**

- A case–control design will yield a high number of patients with and without AF in order to determine operating characteristics with good accuracy and our population will be generalisable.
- Twelve-lead ECGs will be performed on all patients and interpreted blindly by consultant cardiologists. New cases of AF will be detected in the control group and participants will be informed of the diagnosis.
- The use of rate-limiting medication in the case group may influence diagnostic sensitivity.
- The devices will be tested in a nurse-led clinical setting (unsupervised, home use could lead to excess noise or artefact and suboptimal performance).
- The diagnostic algorithm has been optimised for single-use screening and may not perform optimally for prolonged screening (which could be evaluated in future trials).

## Background

Atrial fibrillation (AF) was first identified in 1909 and has assumed increasing importance due to the surges in the elderly population^1^ and the increasing prevalence of the metabolic syndrome.^2^ It is estimated that between 15% and 20% of strokes are AF-related.^3^ Around one in 10 people aged over 65 have AF in the UK.^4^ The lifetime risk for AF is one in four for Americans older than 40 years.^5^ A significant proportion of AF is paroxysmal (25–62%)^6^ and up to 90% of these episodes are asymptomatic.^7^ Approximately 25–40% of ischaemic strokes are cryptogenic and it has been postulated that additional extended electrocardiographic monitoring may identify aetiologic paroxysmal AF in a subset of these strokes.^8^ In poststroke patients, a 30-day period of automatically triggered event recording increased the detection rate of paroxysmal AF fivefold compared with a 24-hour Holter (16.1% vs 3.2%).^9^ In one study, the occurrence of any device-detected AF (lasting only 6 min) in the first 3 months of observation was associated with a 2.5-fold increase in the risk of ischaemic stroke or systemic embolism over a follow-up of 2.5 years.^10^ Anticoagulation treatment using warfarin (or novel oral anticoagulants) is effective in reducing the risk of AF-related stroke by approximately two-thirds and could provide a 10% reduction in overall mortality.^11^

During AF, the atrial activity is disorganised resulting in an irregular ventricular rate which causes irregularity of the RR intervals and the absence of P-waves on an ECG trace^12^ which is required in order to establish the diagnosis. When used as screening tools, however, ECG and ambulatory rhythm recordings are costly and time consuming. In addition, many primary care professionals cannot accurately detect AF on an ECG, and conventional interpretative software is not sufficiently accurate to circumvent this problem, even when combined with interpretation by a general practitioner.^13^

Recently, new medical devices containing diagnostic algorithms have been designed specifically to detect AF such as AliveCor^14^ (a handheld single-lead ECG system using a smartphone) and WatchBP^15^ (a blood pressure metre advocated by the National Institute for Health and Clinical Excellence (NICE)) and these have been shown to be accurate in a meta-analysis.^16^ AliveCor and WatchBP can, however, only measure the ECG or pulse over short time periods and as yet, there is no screening programme for AF in the UK. A recent Cochrane Review has suggested that additional research is needed to examine the effectiveness of alternative screening strategies and the effects of the intervention on risk of stroke for screened versus non-screened populations.^17^ There are also organisational barriers, such as lack of time, staff and capacity, to be overcome for AF screening to be feasibly implemented within primary care.^18^

Inexpensive consumer devices such as heart rate monitors and other wearable technology may have the potential to accurately detect irregular RR intervals^19^ ^20^ and advancements in modern technology have enabled the implementation of relatively complex algorithms using readily available consumer devices including smartphones.^14^ ^21^ Systems employing wearable devices could be used for prolonged RR interval monitoring and may thus have potential for AF screening in the community. We have developed an AF detection algorithm for intended use with devices that detect consecutive RR intervals. The algorithm employs methods described elsewhere in the literature including turning points,^22^ clustering within Lorenz Plots^23^ and Shannon Entropy^24^ and has been tested extensively using publicly accessible sets of clinical data.^25^ Details of the development and optimisation of the algorithm are due to be submitted for publication. We evaluated several consumer devices including wearable ECG-based RR interval recorders and several wearable photoplethysmographic (PPG)-based devices. The PPG devices were subject to excessive noise during prolonged monitoring and we found that supraventricular ectopic beats did not always yield detectable PPG signals (unpublished data) and thus chose to evaluate ECG-based devices which could potentially have superior sensitivity compared with PPG-based techniques.^21^ ^26^

The authors have secured funding from the School of Primary Care Research (SPCR) within the National Institute of Health Research (NIHR) in the UK to investigate the algorithm (using RR intervals sent via bluetooth from a Polar H7 heart rate monitor^27^ and also in an offline setting using RR intervals recorded by the Firstbeat Bodyguard 2 device^20^). Our trial is due to begin recruitment in September 2016. The Screening for Atrial Fibrillation using Economical and accurate TechnologY (SAFETY) trial will investigate if readily available and inexpensive consumer devices can accurately detect AF in general practice patients aged 65 years and above. Additionally, the study will directly compare the accuracy of two existing devices (a handheld ECG system, AliveCor and an automatic sphygmomanometer, WatchBP) alongside the bespoke algorithm. Importantly, we will collect qualitative data from patients regarding their perspectives on using wearable devices and also from GPs regarding their views on AF screening.

## Methods/design

### Objectives

The primary objective is to compare the accuracy of several devices against gold standard (12-lead ECG interpreted by a panel of cardiologists) for the detection of AF:

1. WatchBP;
2. AliveCor;
3. a bespoke algorithm using a polar heart rate monitor belt and smartphone application;
4. a bespoke algorithm using a wearable device (Firstbeat Bodyguard 2).

Secondary objectives will be to use quantitative data to evaluate the participants’ experiences of using the wearable devices and GPs’ perspectives on AF screening.

### Study design

We will perform a multicentre case–control diagnostic study comparing four different methods for detecting AF with a reference standard consisting of a 12-lead ECG. The trial will be conducted in GP surgeries in Hampshire, UK. Participants will be given patient information sheets and the opportunity to ask questions. All participants will give written informed consent prior to participation. Case–control studies have been shown to lead to higher estimates of diagnostic accuracy compared with single series of consecutive patients due to factors such as severity of illness, alternative diagnoses and comorbid conditions.^28^ The proposed study design is unlikely to lead to inflated estimates of diagnostic accuracy as the inclusion criteria for the control group are broad and there is not known to be a temporal effect on the severity of AF or degree of heart rate variability. There will be crossover between the case and control group due to the absence of AF in some of the case group if they have reverted into sinus rhythm or have paroxysmal AF and the detection of new cases of AF in the control group. In addition, the use of rate limiting medication in the case group may reduce heart rate variability and lower diagnostic accuracy in the case group.

#### Participants, practices and patients

General practices will be recruited across Hampshire sequentially and we anticipate between five and 10 practices will be needed. The inclusion criteria are age >65 and can read and speak English. We will select patients with and without pre-existing AF (as recorded in the GP medical records) as per our sample size calculation. In the event of detecting new cases of AF, we will notify the participant and respective GP. Our exclusion criteria are listed below:

- having a pacemaker;
- deemed unsuitable for study by named GP (eg, terminally ill, bedridden);
- lacking capacity;
- previous moderate or severe skin reaction to electrode gel.

#### Sample size

If we assume the specificity of the new algorithm is between 97% and 99%, to detect a difference in specificity compared with the other devices of 5% (ie, 97% vs 92%; 98% vs 93% or 99% vs 94%) requires 329 individuals who do not have AF for 80% power and α of 0.05. Assuming the sensitivity of the devices using the bespoke algorithm is 95% to 99% then 73 individuals with AF are needed to estimate the sensitivity to within ±5%. A recent study found the AliveCor automated algorithm to have a specificity of 99.4% but a relatively low sensitivity of 71.4% and it may be that AliveCor have modified their algorithm.^26^ In this case, we would expect to detect a significant difference in sensitivity between AliveCor and the new algorithm.

We aim to recruit known AF patients (from GP records) to reduce the population size required (although some of these patients may have reverted to sinus rhythm or have paroxysmal AF) and also patients not known to have AF who will represent a typical screening group with a low prevalence. Assuming that 20% of patients coded as AF in the GP records will have paroxysmal AF or not be in AF when they attend, we will increase the numbers in this group accordingly to 92. We also aim to recruit AF and non-AF patients at the same rate and to have the ECGs coded in batches and therefore we can stop recruiting patients with pre-existing AF when we have achieved more than 73 patients coded as AF on their ECG. We will include further subjects not known to have pre-existing AF who are subsequently diagnosed on their ECG.

#### Randomisation

The four devices will be tested on each participant in a random sequence. The sequences for each participant will be generated by the trial statistician.

#### Outcomes

Primary outcome: The primary outcome is to compare the accuracy of four devices against gold standard (12-lead ECG). We will document the sensitivity and specificity with their 95% CIs. The gold standard will be ECG diagnosis of AF by a panel of three cardiologists.

*Secondary outcomes*: We will ask participants to rate comfort and overall impression for each device on a score of 1–10. We will collect qualitative data from participants capturing their experience of using wearable devices in order to evaluate acceptability. We will also collect data from GPs to determine issues relating to their views on AF screening.

#### Recruitment

Wessex Clinical Research Network will identify suitable GP practices and individual participants will be recruited through GP records searches. GPs will subsequently select suitable participants from the searches based on the exclusion criteria and invitation letters will be sent. All participants will be required to give informed written consent prior to participation.

#### Blinding

The study nurses and investigators will be blinded regarding the presence of pre-existing AF. The panel of cardiologists will also be blinded to the presence of known AF. There will also be an expected crossover between groups as some patients coded as having AF may be in sinus rhythm and a proportion of the control group will have AF.

#### Procedures/study schedule

**Figure 1:**
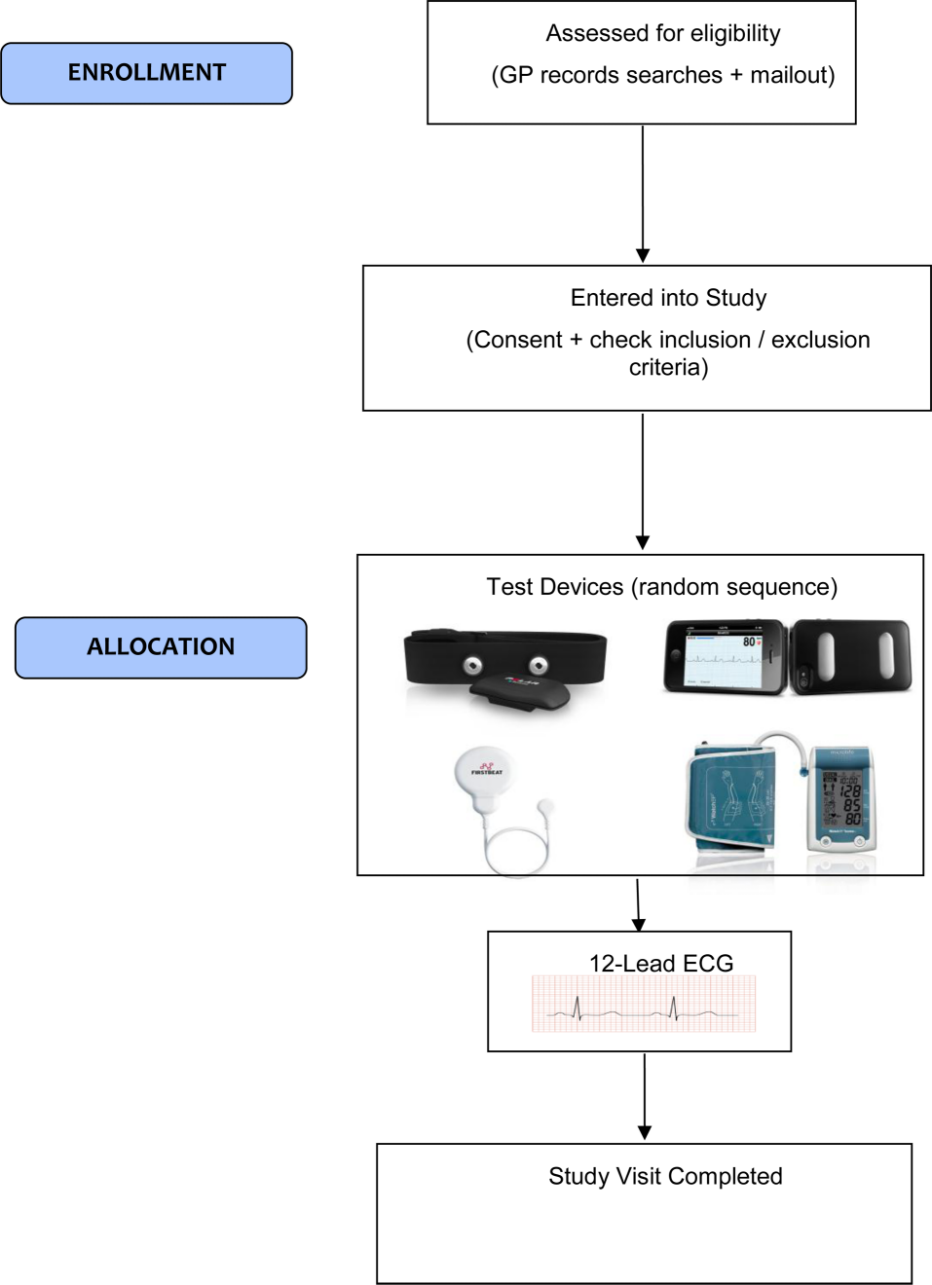
Study visit of Screening for Atrial Fibrillation using Economical and accurate TechnologY (SAFETY)—a pilot study.

The study will be conducted by Good Clinical Practice (GCP) certified researchers. Participants will have an AF detection screen using four devices in randomised order (Polar H7 belt with bespoke algorithm; second wearable device (Firstbeat Bodyguard 2), WatchBP, AliveCor). A randomisation code will be generated by the trial statistician for each participant and list the order to test the devices (eg,1. Polar H7, 2. AliveCor, 3. WatchBP, 4. Firstbeat Bodyguard 2). The time taken for the testing is likely to be around 30–40 min. Although some patients with paroxysmal AF may experience changes in heart rhythm during the visit, we did not want to subject the study population to multiple ECG recordings and single ECG recordings have been used elsewhere.^29^ The ECGs will be reviewed by a panel of three cardiologists as to whether AF is present. The study visit is depicted in figure 1.

Technical issues surrounding the use of the equipment will be addressed by the research nurse. For example, if the operation of a device is not initially successful or an ECG is of poor quality, further recordings may be attempted as long as this is deemed reasonable and acceptable by the nurse and participant in question. A reasonable number of attempts may be made to fit the polar belt and obtain readings, reuse AliveCor if data are not obtained and to repeat the WatchBP measurement. The number of attempts required will be recorded. If a reading is not obtained using a device, this will be recorded along with a brief description of the problem(s) encountered. We will record the overall classification for each device as AF/not AF (the WatchBP Home S device used in the trial records a minimum of three measurements). The device testing is detailed in table 1.

**Table 1:** Device testing characteristics

	Minimum time taken for a single recording/usage	Minimum number of readings	Reading to be used in trial	ECG trace/RR intervals/peripheral pulse measurement
AliveCor	30 s	1	1st complete 30 s reading	ECG trace
WatchBP	∼3.5 min	3	1st complete set of three readings	Peripheral pulse
Polar H7	45 s	1	1st completed reading	RR intervals
Firstbeat Bodyguard 2	2 min	1	1st completed reading	RR intervals

We anticipate that research nurse-led testing will lead to a reduction in artefact/noise and thus potentially improve diagnostic accuracy compared with unsupervised use of the devices. In particular, we anticipate optimal quality of AliveCor/single-lead ECG recordings with nurse-led testing and similarly with nurse-led blood pressure measurements. Although nurses will fit the Polar H7 heart rate monitor belt, this device is designed to detect RR intervals during vigorous activity and is therefore likely to be relatively insensitive to noise. Similarly, the Firstbeat Bodyguard 2 device is an RR interval recorder that uses strongly adhesive gel electrodes and is designed for accurate use during periods of vigorous activity.

The data used by the bespoke algorithm (RR intervals) will be stored on the paired device (iPad). The data will be anonymous and contain a patient ID number, time and date information and a list of RR intervals along with the overall classification (suspected AF/not AF). Offline analysis can be performed to verify the algorithm and the data set could potentially be used for further algorithm development. The AliveCor readings will be stored using patient ID only. In this trial we will evaluate a single successful use of each device and will not be evaluating AF detection with prolonged screening. The algorithm we will use has been optimised for single-use screening. It is likely that prolonged screening could generate more false positive results. Significant advancements in the technology of consumer devices may enable periods of ECG data to be stored or transmitted via Bluetooth for review in the future which could be used for clinician review of suspected AF during prolonged screening which we would hope to evaluate in the future. The Polar H7 and Firstbeat Bodyguard 2 devices do not currently store or transmit continuous ECG data.

The software code used to implement the bespoke algorithm will be printed, signed and stored in the site file. The firmware and algorithm versions of the AliveCor and WatchBP algorithms will also be stored in the site file and we will continue to use the same versions throughout the trial. In this trial we will evaluate the diagnostic performance of the device algorithms without clinician input. It should be noted that the AliveCor device has ‘unreadable’ and ‘unclassified’ diagnostic categories and we will record these but only include ‘suspected AF’ classification as positive results in the main analysis and perform a subgroup analysis excluding the unreadable and unclassified results.

### Qualitative data

We would like to evaluate the acceptability of these devices among clinicians who may use and recommend them and patients who may wear them for extended periods. We will aim to recruit 15–25 individuals who do not have AF and provide them with the wearable devices to evaluate for prolonged use, and will also interview between 10 and 15 GPs and practice nurses. Purposive sampling will be applied to ensure broadly similar representation of men and women and individuals from urban and rural settings. Semistructured interviews will be conducted using focus groups with digitally recorded, transcribed verbatim and identifiable data removed for ethical reasons. Inductive thematic analysis of each transcript will be carried out. Deviant case analysis will be used to ensure that perspectives that diverged from dominant trends are not overlooked.

The initial coding structure will be revised to develop a coding manual based on consensus among the research team. This will result in a robust conceptual overview of the factors that influence patients to use the various AF monitors being evaluated and whether they can be self-applied and used for continuous periods of 24 or 48 hours reliably and with comfort. This will allow us to understand the pragmatic issues around the use of each of the appliances and will substantively influence further evaluation of this equipment for the detection of AF in primary care. It will allow us to address our feasibility objectives and translate this study into more definitive and patient centred recommendations about the use of this equipment in the community. It will also allow us to understand the issues that may face clinicians in primary care when recommending the use of this equipment to screen and diagnose.

### Statistical analysis

We will collect baseline data including age and gender. We will document the sensitivity and specificity with their 95% CIs. We will also test whether the specificity is significantly greater using the bespoke algorithm by comparing the proportions using the χ^2^ test. The gold standard will be ECG diagnosis of AF by a panel of three cardiologists. The ECGs will be analysed in batches in order that new AF diagnoses are conveyed during the trial. If a subject is not known to have pre-existing AF and is diagnosed by ECG as having AF, we will send a copy of the ECG to the GP and also inform the patient and include a patient information leaflet on treatment options.

## Discussion

The SAFETY trial will be one of the first trials to directly compare two existing AF screening devices (with NICE recommendation) within the same trial. In addition, this will be the first trial to evaluate inexpensive wearable consumer devices that can be used for prolonged monitoring and directly compare these with both existing AF detection devices and gold standard 12-lead ECG. Importantly we will also collect quantitative and qualitative data from the trial participants to evaluate the suitability of the devices. We have chosen participants aged 65 and above as this will yield a higher prevalence of previously undetected AF and the over 65s are at higher risk from AF-related stroke. In addition, we will gain insights from GPs regarding AF screening including their opinions on the devices, and wider issues such as workload. Based on the results, we will aim to design a larger trial to investigate prolonged AF screening in the community using inexpensive consumer devices.

Trial Status

We began recruitment in November 2016.
